# Rationale and development of a business case for antimicrobial stewardship programs in acute care hospital settings

**DOI:** 10.1186/s13756-018-0396-z

**Published:** 2018-08-29

**Authors:** A. M. Morris, E. Rennert-May, B. Dalton, N. Daneman, L. Dresser, S. Fanella, J. Grant, Y. Keynan, N. Le Saux, J. McDonald, Y. Shevchuk, D. Thirion, J. M. Conly

**Affiliations:** 1 Department of Medicine, University of Toronto, Sinai Health System, and University Health Network, Toronto, ON Canada; 2 Department of Medicine, University of Calgary and Foothills Medical Centre, Alberta Health Services, Calgary, AB Canada; 3 Pharmacy Services, Foothills Medical Centre, Alberta Health Services, Calgary, AB Canada; 4 Department of Medicine, University of Toronto and Sunnybrook Health Sciences Centre, Toronto, ON Canada; 5 Antimicrobial Stewardship, University Health Network, Toronto, ON Canada; 6 Department of Pediatrics and Child Health and Medical Microbiology, University of Manitoba, Winnipeg, MB Canada; 7 Department of Pathology and Laboratory Medicine, Vancouver General Hospital, Vancouver, BC Canada; 8 Departments of Internal Medicine, Medical Microbiology and National Collaborating Center for Infectious Diseases, University of Manitoba, Winnipeg, MB Canada; 9 Department of Pediatrics, University of Ottawa and Children’s Hospital of Eastern Ontario, Ottawa, ON Canada; 10 Pharmacy Services, Children’s Hospital of Eastern Ontario, Ottawa, ON Canada; 11 College of Pharmacy and Nutrition, University of Saskatchewan, Saskatoon, SK Canada; 12 Faculté de pharmacie, Université de Montréal, Department of Pharmacy, McGill University Health Centre, Montréal, QC Canada; 13 Departments of Medicine and Immunology, Microbiology & Infectious Diseases, University of Calgary and Alberta Health Services, AGW5 Ground Floor SSB, Foothills Medical Centre, 1403 29 St NW, Calgary, AB T2N 2T9 Canada

**Keywords:** Antimicrobial stewardship, Antimicrobial resistance, Business case, Human resources

## Abstract

**Background:**

Antimicrobial stewardship programs (ASPs) have been shown to reduce inappropriate antimicrobial use and its consequences. However, these programs lack legislative requirements in many places and it can be difficult to determine what human resources are required for these programs and how to create a business case to present to hospital administrators for program funding. The objectives of the current paper were to review legislative requirements and outline human resource requirements for ASPs, and to create a base business case for ASPs.

**Methods:**

A working group of antimicrobial stewardship experts from across Canada met to discuss the necessary components for creation of a business case for antimicrobial stewardship. A narrative review of the literature of the regulatory requirements and human resource recommendations for ASPs was conducted. Informed by the review and using a consensus decision-making process, the expert working group developed human resource recommendations based on a 1000 bed acute care health care facility in Canada. A spreadsheet based business case model for ASPs was also created.

**Results:**

Legislative and /or regulatory requirements for ASPs were found in 2 countries and one state jurisdiction. The literature review and consensus development process recommended the following minimum human resources complement: 1 physician, 3 pharmacists, 0.5 program administrative and coordination support, and 0.4 data analyst support as full time equivalents (FTEs) per 1000 acute care beds. Necessary components for the business case model, including the human resource requirements, were determined to create a spreadsheet based model.

**Conclusions:**

There is evidence to support the negative outcomes of inappropriate antimicrobial use as well as the benefits of ASPs. Legislative and /or regulatory requirements for ASPs are not common. The available evidence for human resource recommendations for ASPs using a narrative review process was examined and a base business case modelling scenario was created. As regulatory requirements for ASPs increase, it will be necessary to create accurate business cases for ASPs in order to obtain the necessary funding to render these programs successful.

**Electronic supplementary material:**

The online version of this article (10.1186/s13756-018-0396-z) contains supplementary material, which is available to authorized users.

## Background

Antimicrobials are medications used for the treatment of infectious diseases and their development and use in the twentieth century has contributed substantially to a decline in infectious disease-related mortality [1]. However, the success of these medications in curing illnesses has been associated with adverse events and unintended consequences [2]. As many as one in five visits to the emergency room related to adverse drug reactions are due to antimicrobials [3]. Diarrhea is a common adverse event and *Clostridium difficile* infection (CDI) may occur in up to 20% of cases of antibiotic-associated diarrhea [4]. Other harmful effects of antimicrobials include rashes, allergies, organ damage (e.g. the kidneys and liver), effects on the bone marrow, and drug interactions [3]. Antimicrobial overuse is considered the principal driver in selecting for antimicrobial-resistant organisms with a strong association between rising rates of use and increasing rates of antimicrobial resistant organisms in both inpatient and ambulatory settings [5–9].

Against this background of rising rates of antimicrobial resistant organisms and serious adverse events associated with antimicrobial use has been the drive to develop antimicrobial stewardship programs (ASPs) with the primary intent of reducing inappropriate antimicrobial use. There is growing evidence demonstrating the benefit of antimicrobial stewardship initiatives on both process measures such as antimicrobial consumption, and on outcome measures including antimicrobial resistance and length of hospital stay [10, 11].

However, despite many international organizations providing generic recommendations for human resources requirements for ASPs, no groups have reviewed if any regulatory and/or legislative requirements have been provided within any specific jurisdictions or outlined a detailed human resources rationale or created a specific business plan that may be used to justify the need for such programs to hospital or regional administration. A group of experts in antimicrobial stewardship from across Canada met to review the rationale for ASPs with a focus on the Canadian landscape and sought to address the following: review relevant organizational mandates or legislative requirements for ASPs, develop a sound rationale for the human resource requirements for ASPs, and create a business case that could be presented to health care administrators to support the rationale for the development of an ASP.

## Methods

### Literature review of regulatory requirements and human resources recommendations for antimicrobial stewardship programs

An Association of Medical Microbiology and Infectious Diseases (AMMI) Canada Working Group, comprised of antimicrobial stewardship multidisciplinary experts (infectious diseases physicians, pharmacists, and microbiologists) from across Canada, convened in Ottawa, Ontario in September 2016 and focused on the following key areas: relevant regulatory and legislative requirements for ASPs with a focus on Canadian requirements and a process for determining the human resources personnel and their respective full time equivalents (FTE) to manage an effective ASP within the acute care hospital setting.

The Working Group used a narrative style literature review and surveyed the relevant literature on this topic through searches in PubMed and Medline (English and French language only) and reviewed the analogous evidence base in infection control with respect to FTE requirements. A list of search terms utilized can be found in Additional file 1: Appendix A. The references of any identified relevant papers were searched as well and the Working Group experts also suggested specific references of which they were aware which were cross-referenced against the list of search terms. Where appropriate, relevant articles were appraised using tools relevant to the types of studies according to the Cochrane Collaboration [12] and the study quality appraisal checklists of the National Institute for Health and Clinical Excellence (NICE) in the United Kingdom [13]. The Working Group then developed ASP human resource recommendations based on a 1000 bed acute care health care facility in Canada using a consensus decision-making process. Consensus was achieved through iterative discussion following the review of the relevant articles. No members of the Working Group had any conflicts of interest with respect to the consensus decision-making process.

### Development of a base business case modelling scenario

The Chair of the Working Group developed a spreadsheet (Excel Version 10.0 Microsoft Corp., Redmond, Washington) which may be used to populate the rates of relevant untoward events (antimicrobial resistant organisms and CDI) and FTE requirements for an individual acute health care institution. The model program was reviewed by the Expert Working Group.

## Results

### Review of regulatory and legislative requirements

The literature review found only two references related to regulatory and legislative requirements at the national level. ASPs were incorporated as a Required Organization Practice by Accreditation Canada and are a required component in acute care hospitals since January 2013 [14]. The required organization practice suggested multiple possible interventions including prospective audit and feedback, formulary of targeted antimicrobials and approved indications, education, guidelines and clinical pathways, antimicrobial order forms, dose optimization and parenteral to oral conversion [14]. In addition, antimicrobial stewardship was included in the National Safety and Quality Health Service Standards required for hospital accreditation in Australia in 2012 [15]. The only other jurisdiction for which we identified a similar organizational mandate to have multidisciplinary ASP at the time of consensus development was the state of California, where legislation (California Senate Bill 1311) was passed in September of 2014. This bill required hospitals to “develop a physician supervised multidisciplinary antimicrobial stewardship committee and to appoint at least one physician or pharmacist to that committee, subcommittee, or workgroup who is knowledgeable about antimicrobial stewardship through prior training or attendance at continuing education programs [16].” The Joint Commission in the United States did address antimicrobial stewardship and a new medication management standard for hospitals, critical access hospitals and nursing care centers, and this became effective following the time of consensus development on January 1, 2017 [17]. Study quality assessment tools were not considered appropriate to be applied to these articles.

### Human resources recommendations for antimicrobial stewardship programs

Our search found generic suggestions for what human resources are required for ASPs but very few published studies which described the detailed FTE positions that may be required and none provided an evidence based rationale for the FTE requirements. A single study of a cross-sectional survey of healthcare facilities (*n* = 65) from France concluded that 3.6 FTE leads, 2.5 FTE pharmacists and 0.6 FTE of dedicated microbiologist time per thousand beds were required to implement and sustain ASP activities [18]. Study quality assessment suggested that although the questionnaire design, survey and analysis of responses was adequate the results have considerable uncertainty given that they represent only 2.3% (65/2480) of all the public and private healthcare facilities in France in 2013 and representativeness is a major bias. Recent articles which were published following the consensus development process provided expert opinion commentary on the need to provide human resource requirements for ASP teams with protected time for key personnel and produced a table summarizing country level recommendations for antibiotic stewardship teams in acute care hospitals drawn from various professional or public health organization websites [19, 20].

In the acute care hospital setting there are analogous recommendations for FTE requirements for infection control programs and some evidence to support them. We were able to draw parallels with the infection control staffing requirements which may be extrapolated to ASPs given the similarity between the programs and the workforce required. The scientific basis for claims of efficacy of hospital infection control programs was established by the Study on the Efficacy of Nosocomial Infection Control (SENIC) project [21]. This study found “a trained hospital epidemiologist to be an essential component of an effective hospital infection control program” and suggested that one infection control practitioner (ICP) per 250 occupied beds was associated with an effective program [21].

Staffing requirements of ICPs were reviewed in the United States using a Delphi process and published recommendations are for a ratio of 0.8–1.0 FTE ICPs per 100 occupied acute care beds [22]. In Canada, the Public Health Agency of Canada’s Infection Control Guidelines Steering Committee developed the “Essential Resources for Effective Infection Prevention and Control Programs: A Matter of Patient Safety” [23] to determine what human resources were needed in order to deliver effective infection prevention and control programs. The findings suggested three ICPs per 500 beds in an acute-care facility and one ICP per 150–250 beds in a long-term care institution [23].

Based on the very limited findings from the literature review of FTE requirements for ASPs and the accepted infection prevention and control staffing requirements which may be readily extrapolated to ASPs, the Working Group developed acute care hospital ASP staffing recommendations. Their suggestions were based on a 1000 bed hospital, including intensive care units and all medical and surgical specialties, but no transplant services (Table 1). The recommendations were considered relevant to individual acute care hospitals, a health system or collection of hospitals, from a Canadian perspective. These recommendations will require adjustment based on the complexity of patients treated within the specific site. For example, sites with burn centres, bone marrow or organ transplantation may require a more intensive ASP while a rehabilitation centre may require a less intense ASP.

**Table 1:** Recommendations for stewardship human resources personnel in an acute care hospital setting in Canada

Personnel	Full time equivalents per 1000 acute care beds
Physician	1.0
Pharmacist	3.0
Project/Program Administrative and Coordination Support	0.5
Data Analyst	0.4

### Base business case modelling scenario

In the business case modelling scenario, a generic template developed in Excel was designed to allow Business Managers, ID physicians, Infection Control staff, and pharmacists to project the return on investment from the implementation of an antimicrobial stewardship program (ASP). Data requirements for the use of the template requires input of the following:1) personnel, start-up and antimicrobial costs incurred in the planning and development of the ASP and 2) ASP FTE requirements, programmatic and antimicrobial costs incurred to support the on-going operation of the ASP.

Costs to consider included personnel costs (ASP physician, pharmacists, project manager, data analyst, and data entry; antimicrobial costs which should be included at baseline, with projected increases without an ASP and decreases with an ASP and; the potential indirect cost savings (cost avoidance) associated with a reduction of CDI cases. Operational costs including office operations and equipment (e.g. computers and software) are also needed. A ‘variables’ worksheet was suggested to be provided to the user with most important variables, as well as the incremental analysis. The generic template is available in an open access format on the AMMI Canada website [24]. The generic model was not tested or validated against any specific healthcare facility in Canada and was not proposed to any health care administrators prior to its development. Its major purpose was to use it as a “tool” into which different inputs could be inserted to build scenarios for presentation to hospital administration.

## Conclusions

Hospital antibiotic prescribing is driven by many complex influences, including the perceived attempt to maximize care for individual patients. In many cases, such as CDI, not only will an individual suffer the effects of this adverse event, but these infections may also spread to close contacts, within the hospital but also throughout the community. Healthcare facilities have a crucial role and obligation to manage, oversee and provide access to tools for physicians and other prescribers to optimize the use of antimicrobials. Accountability also rests with hospital administrators to ensure that appropriate ASPs are in place. Antibiotic resistance is a threat to public health around the world. A concerted effort by healthcare institutions to lead the way in the management of existing antimicrobials will help stem global increases in antimicrobial resistance. There is an urgent need for hospital administration to encourage and support ASP initiatives to enhance appropriate antimicrobial use and evaluate antimicrobial use across institutions.

Collectively, the resources described above, including the regulatory requirements, FTE rationale for personnel and the generic case modelling scenario template provide the key elements for a “business case” to be presented to hospital administrators for the development of their institutional ASPs. However, any business plan requires modifications based on the size of the institution, the patient population it serves, and the location (e.g. urban versus rural). There needs to be recognition that there will be many other programs and departments putting forth business cases to hospital or regional administrators and that not every program can be funded [25]. These points were emphasized in the only other article we were able to find on the “business pitch”.

The business plan must address any anticipated costs and potential cost-avoidance for the institution, and the business case analysis as mentioned previously can aid in developing this component. While necessary to ensure that costs and the potential for cost-effectiveness are addressed, it is important to emphasize that the main focus of ASP is patient safety and reduction in inappropriate antimicrobial use. Additionally, if there are cost savings initially (e.g. through reduced antimicrobial expenditures, less adverse events such as CDI or shorter hospital stays), with time these savings may disappear as an ASP requires a constant input of resources, however these programs can still be cost-effective. More members of a team including physicians and pharmacists means that more interventions can be undertaken leading to a greater impact. This should be explained in detail so that the costs of the personnel can be warranted. Finally, engaging decision makers early in the process is imperative. This allows time to develop a relationship and credibility.

We recognize there are limitations to our findings including the very limited numbers of references to FTE requirements that we were able to find, the use of a narrative review process with a more limited search strategy than a systematic review, the difficulty in finding grey literature on business plans for ASPs, the use of extrapolation from the requirements for ICPs which may not be directly applicable to pharmacists and the limitation to an acute care setting. The findings are also applicable to a Canadian perspective and may not reflect other higher income countries and would not be applicable to lower and middle income countries where resources may be more limited. Given the relative dearth of published literature on the topic of FTE requirements, our findings may be considered more of an expert opinion but we believe the rationale is sound.

Nonetheless, we believe the findings presented will add to the growing literature on ASPs and our process was original with respect to pursuing the evidence base for the FTE requirements and using an extrapolation method from accepted human resources requirements for infection prevention and control programs in Canada and the United States. Additionally, a novel scenario template was created to allow others to build their business cases in a credible and meaningful manner for presentation and discussion with hospital and regional healthcare administrators. We believe the template could be modified to include specific quality indicators for appropriate antibiotic use [26] and also to be applicable to lower and middle income country settings.

## Additional file

Additional file 1: **Appendix A.** List of search terms for legislative requirements and human resource staffing requirements in antimicrobial stewardship programs. (DOCX 18 kb)

## Abbreviations

AMMI: Association of Medical Microbiology and Infectious Diseases Canada
ASPs: Antimicrobial Stewardship programs
CDI: *Clostridium difficile* infection
FTE: Full time equivalents
ICP: Infection control practitioners
