# Supplementary material for: Rationale and development of a business case for antimicrobial stewardship programs in acute care hospital settings
Source: Antimicrob Resist Infect Control. 2018 Aug 29;7:104. doi: 10.1186/s13756-018-0396-z (PMC6114185; doi:10.1186/s13756-018-0396-z)
Supplement: Supplementary file 1 — Appendix A. List of search terms for legislative requirements and human resource staffing requirements in antimicrobial stewardship programs. (DOCX 18 kb) [file 13756_2018_396_MOESM1_ESM.docx]

**Additional File 1**

Appendix A

List of search terms for legislative requirements and human resource staffing requirements in antimicrobial stewardship programs:

antimicrobial stewardship and Canadian Standards

Regulatory

Examples

antimicrobial stewardship and International Standards

Regulatory

Examples

microbiology

Support and resources

Expenditures

Infection Prevention and Control

Support and resources

Policies re: antimicrobial resistant organisms

Consequences of antimicrobial resistance (e.g. isolation bed-days)

antimicrobial stewardship proposal(s)

resources antimicrobial stewardship

Human (salary and in-kind, including benefits where appropriate)

Office Operations

Equipment

Antimicrobial essential componen
